# Engineering a nanolab for the determination of lysosomal nitric oxide by the rational design of a pH-activatable fluorescent probe†
†Electronic supplementary information (ESI) available. See DOI: 10.1039/c5sc04415d


**DOI:** 10.1039/c5sc04415d

**Published:** 2015-11-30

**Authors:** Yinhui Li, Wei Wu, Jinfeng Yang, Lin Yuan, Changhui Liu, Jing Zheng, Ronghua Yang

**Affiliations:** a State Key Laboratory of Chemo/Biosensing and Chemometrics , College of Chemistry and Chemical Engineering , Hunan University , Changsha , 410082 , China . Email: yinhuili16@163.com ; Email: yangrh@pku.edu.cn; b School of Chemistry and Biological Engineering , Changsha University of Science and Technology , Changsha , 410004 , China; c Tumor Hospital , Xiangya School of Medicine , Central South University , Changsha , 410013 , China

## Abstract

Nitric oxide (NO) is often involved in many different physiological processes including the regulation of lysosomal functions. However, it remains a great challenge to explore the variations of NO levels in lysosomes, limiting the understanding behind its biological functions in cellular signaling pathways and various diseases. Herein, a pH-activatable fluorescent probe, **Rhod-H-NO**, was designed and synthesized for the determination of lysosomal NO, in which the activation response model is beneficial towards getting accurate biological information. To ensure that **Rhod-H-NO** can accumulate effectively and exist stably in lysosomes without interference and degradation from other active species, **Rhod-H-NO** was engineered into the nanopores of mesoporous silica nanoparticles (MSNs) with β-cyclodextrin (β-CD) as the gatekeeper to obtain a nanolab. The nanolab was successfully applied to detect lysosomal NO in living cells and *in vivo* with high time and spatial resolution. This nanolab could serve as an excellent molecular tool to exploit and elucidate the function of NO at sub-cellular levels.

## Abstract 2

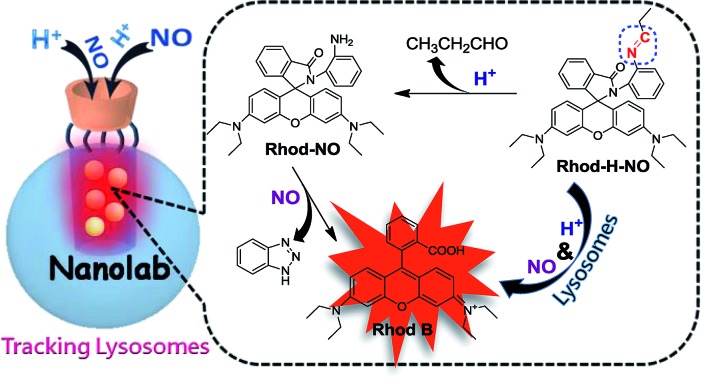
A pH-activatable fluorescent probe, **Rhod-H-NO**, was designed and synthesized for the determination of lysosomal NO in living cells and *in vivo*.

## Introduction

Nitric oxide (NO), a small uncharged free radical, which is one of the mediators of cellular responses, produced by nitric oxide synthases, plays a very important role as a signaling molecule in a variety of physiological and pathological processes that take place in the cardiovascular, nervous, and immune systems.^1^ In a recent study, it was discovered that lysosomal functions are subtly regulated by NO.^2^ These functions include the degradation of a cells own components to supply energy and nutrients for its growth through the lysosomal machinery,^3^ and can cause various diseases (such as Fabry, Gaucher, or Danon diseases) due to lysosomal disorders.^4^ Recently, numerous fluorescent probes for intracellular NO have been developed and applied to intracellular sensing and imaging,^5^,^6^ which has significantly enriched our knowledge about NO homeostasis and the crucial roles of NO in many biological processes. It is worth noting that obtaining convincing evidence about the interrelation between the variations of NO levels in lysosomes and different physiological processes remains a challenge, because available probes for the selective tracking of NO in the lysosomes of living cells have rarely been reported hitherto. One of the bottlenecks is the lack of organelle-specificity, which results in a high background signal from cytosol and other organelles, meaning that the probes cannot provide high spatial resolution in lysosomes. To tackle this challenge, Xiao *et al.* conjugated a lysosomal-specific morpholine moiety as the guiding unit with a NO probe to target the subcellular organelle recently,^7^ but this strategy suffers from a serious problem: the morpholine moiety exhibits an alkalizing effect on the lysosomes so that longer incubation times with these probes can induce an increase in lysosomal pH and result in cell death.^8^ In addition, existing research shows that organic probes are susceptible to acidic pH and interferences from other intracellular species, and are even prone to being degraded by substantial hydrolase in lysosomes,^9^ which makes the detection unsatisfactory for application to living cells. Therefore, the development of an ideal fluorescent probe with lysosomal targeting is highly desirable for quantifying the variations of NO levels in real time and in a dynamic range of concentrations.

Mesoporous silica nanoparticles (MSNs), popular small-molecule drug reservoir systems which show excellent biocompatibility, ease of functionalization and are nontoxic to cells, have attracted widespread interest for drug delivery purposes within the past decade.^10^ In particular, MSNs can undergo cellular uptake into acidic lysosomes by endocytosis when the nanoparticles are below 200 nm in diameter,^11^ which could provide a potential way to study lysosomal tracking and explore its role in invasion with high-resolution spatial images. Recently, we have developed two fluorescent probes for the detection of lysosomal H_2_S and Cu^2+^ based on simultaneous target and location activation.^12^,^13^ One of the targeting strategies is *via* entrapping the fluorescent probe into the nanopores of MSNs. The results were inspiring to us, showing that the intact probe molecule can be stored in the nanopores without interference and degradation and then automatically accumulated in lysosomes, which successfully realized the detection of lysosomal Cu^2+^ and also provided an efficient method to address these challenges in the determination of lysosomal NO.

**Scheme 1:**
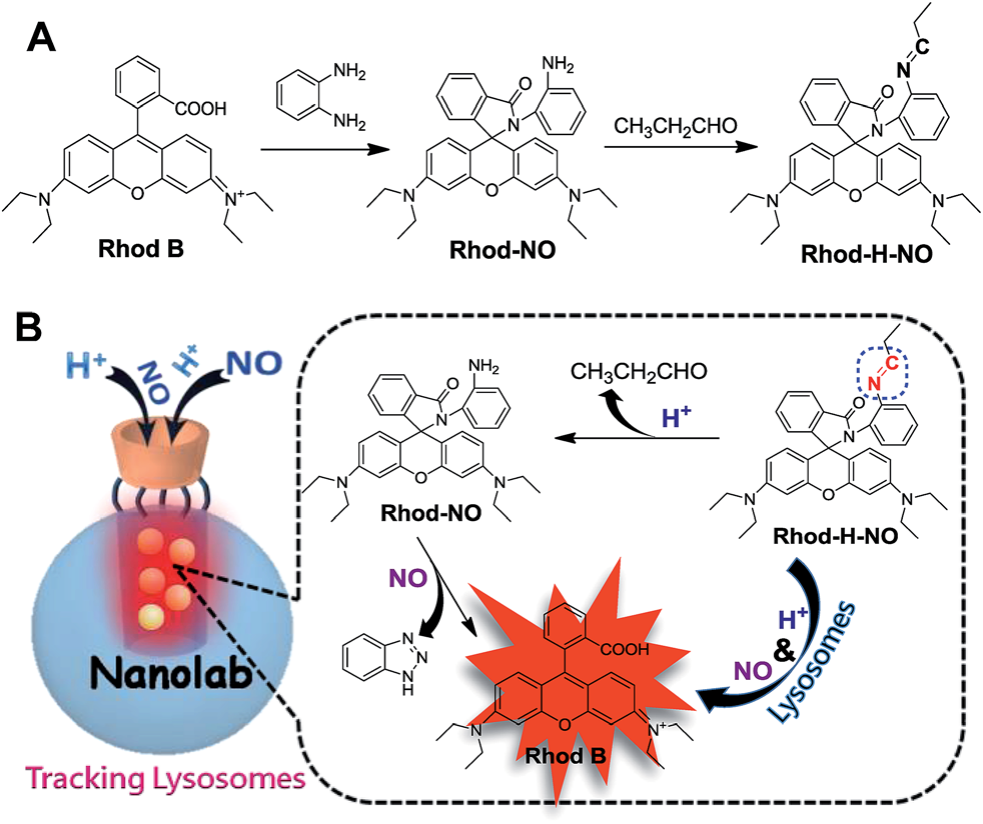
(A) Synthetic route of **Rhod-H-NO**. (B) Schematic illustration of the fluorescence response of **Rhod-H-NO** to NO in the nanolab.

As a part of our ongoing interest in fluorescent probes for application in lysosomes, we herein fabricate a nanolab for the determination of lysosomal NO by engineering a pH-activatable fluorescent probe into the nanopores of MSNs (Scheme 1). Firstly, a pH-activatable fluorescent probe, namely **Rhod-H-NO**, was designed and synthesized for the determination of NO. To avoid the unpredictable fluorescence signals from other organelles and to make full use of the acidic conditions of lysosomes, we adopted a strategy to lock the widely applied NO recognition site moiety of *o*-phenylenediamine by a pH-sensitive imine bond.^14^ The presence of the imine bond shows a silent fluorescence response to NO in neutral and alkaline conditions, while the lysosomal pH (4.0–6.0) of which mediates hydrolysis of the imine group affords a large, rapid, NO-induced fluorescence response. Then, in order to realize the accumulation of probes in lysosomes, MSNs were applied as protective nanocoats to entrap **Rhod-H-NO** in the nanopores, which prevented the probes from interference and degradation, and thus provided a reaction lab for the determination of NO. Finally, β-cyclodextrin (β-CD), a widely reported “gatekeeper” for closing the gates of the pores of MSNs,^15^ was conjugated on the surface of the MSNs to stop external species. This strategy holds the promise of real application in biological research and medical diagnosis.

## Results and discussion

**Rhod-H-NO** was synthesized according to Scheme 1A. The reaction between rhodamine B and *o*-phenylenediamine led to the spiro compound **Rhod-NO**, and further condensation reaction between **Rhod-NO** and propionic aldehyde afforded the probe **Rhod-H-NO** in 65.2% yield. The purified product was fully characterized by NMR spectroscopy and ESI-MS to confirm the structure (Fig. S1–S3†). With the probe **Rhod-H-NO** in hand, we firstly verified the feasibility of the above-mentioned design that NO couldn't “switch-on” the fluorescence of **Rhod-H-NO** unless being activated by acidic pH. As shown in Fig. S4,† **Rhod-H-NO** exhibits no absorption features in the visible region and is essentially non-fluorescent in neutral aqueous solution (pH = 7.0) owing to the spirolactam structure. Upon the addition of NO, there is no significant absorption or fluorescence change indicating that the spirolactam structure remained intact. When **Rhod-H-NO** was treated with an acidic buffer (pH = 5.0), a strong absorption band centered at 552 nm (*ε* = 6.50 × 10^4^ M^–1^ cm^–1^) and a bright fluorescence emission with peak maximum at 590 nm (*φ*_F_ = 0.63) were observed obviously, indicating rhodamine ring-opening *via* a NO-induced reaction in acidic medium. HPLC analysis further demonstrated that the reaction of **Rhod-H-NO** and NO in acidic medium was identical with the proposed mechanism (Fig. S5†). Based on this, we anticipate that the activation response model is beneficial towards fabricating an effective lysosome-targetable molecular tool for exploring NO biology.

**Scheme 2:**
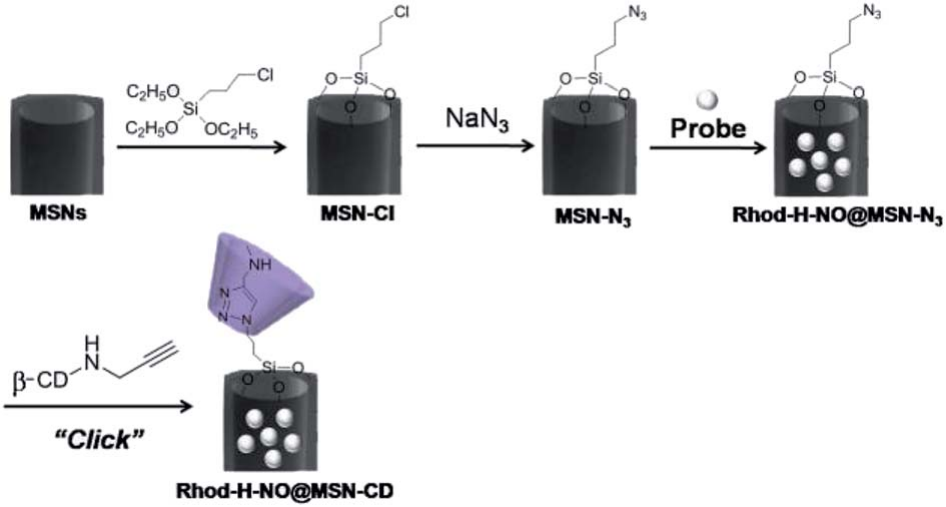
The fabrication process of nanolab from MSNs to **Rhod-H-NO@MSN-CD**.

To ensure that **Rhod-H-NO** can accumulate effectively and exist stably in lysosomes without interference and degradation, a novel nanolab was fabricated by engineering the probe into the nanopores of MSNs and using β-CD as the gatekeeper of the nanopores, which allows NO to pass through the cavities of β-CD and enter the nanopores to react with **Rhod-H-NO**. The fabrication process is shown in Scheme 2. MSNs with typical MCM-41 hexagonal arrangements were first prepared according to the literature protocol.^16^ Then the surface of the MSNs was functionalized with chloride groups by treatment with (3-chloropropyl)triethoxysilane to afford MSN-Cl. The azide terminated MSN-N_3_ was obtained from the reaction of as-prepared MSN-Cl and sodium azide. After removing the surfactant template (*n*-cetyltrimethylammonium bromide, CTAB) from MSN-N_3_, **Rhod-H-NO** was then entrapped in MSN-N_3_ by a diffusion experiment. Subsequently, the CD-alkyne, which was synthesized according to a previously reported method (Fig. S6†),^17^ was attached to the MSN surface using a click chemistry approach, giving the nanolab **Rhod-H-NO@MSN-CD**.

**Fig. 1:**
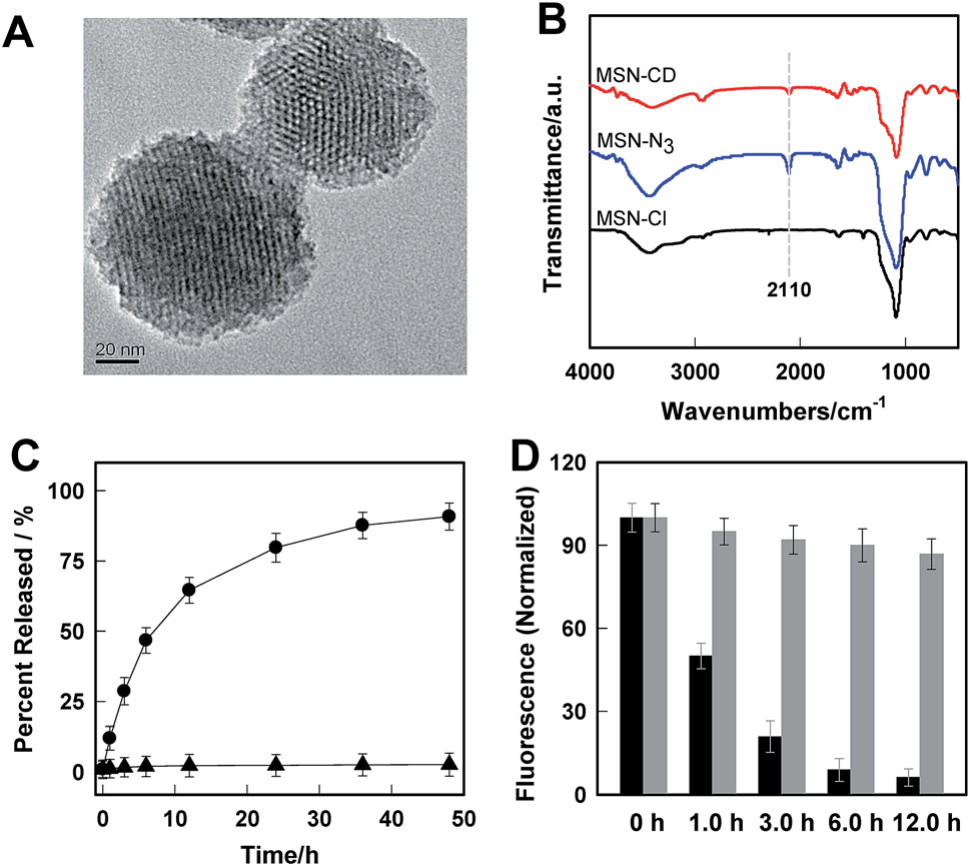
(A) TEM image of MSN-CD. (B) FTIR spectra of MSN-Cl, MSN-N_3_ and MSN-CD. (C) The cumulative release profiles of **Rhod-H-NO** from MSNs () and MSN-CD (▲) in a buffer solution (20 mM, pH = 5.0). (D) The long-term stability of **Rhod-H-NO** (black bar) and **Rhod-H-NO@MSN-CD** (grey bar) in cell lysate.

Transmission electron microscopy (TEM) images showed an obvious border after β-CD was conjugated to the surface of the MSNs, but the average diameter did not show a significant difference (∼80 nm) (Fig. 1A). In addition, MSN-Cl only showed the silica framework vibrations, whereas MSN-N_3_ exhibited the characteristic azide stretch signal at 2110 cm^–1^, but this band was strongly reduced in intensity upon modification with β-CD (Fig. 1B). The zeta potential values of MSN-Cl, MSN-N_3_ and MSN-CD were +18.5 mV, –20.6 mV, and +30.2 mV respectively (Fig. S7†). These results confirmed that the surface of MSNs was well modified with β-CD. When **Rhod-H-NO** was loaded into the nanopores of MSN-CD, XRD patterns showed that the low-angle reflections indexed as (110) and (200) had disappeared (Fig. S8†), and nitrogen adsorption–desorption isotherms and pore size distributions showed that the average pore size decreased with an increase in surface density and infilling (Fig. S9†). Fig. 1C further demonstrates that **Rhod-H-NO** is effectively entrapped in the nanopores without being released for more than 48 h, which is attributed to the size of **Rhod-H-NO** (1.84 nm × 1.29 nm × 0.96 nm, calculated by Gaussian 09 programs) which is larger than the nanopore gates after modification with β-CD. The loading efficiency of **Rhod-H-NO** in MSN-CD was estimated to be ∼15.6 wt%. Owing to the protective effect of the MSNs and the enhanced water-solubility of β-CD, **Rhod-H-NO@MSN-CD** exhibited an improved long-term stability without degradation in cell lysate compared with free **Rhod-H-NO** (Fig. 1D). Most importantly, NO molecules could freely diffuse into the nanolab through the cavities of the β-CD rings to react with **Rhod-H-NO** (Fig. S10†).

**Fig. 2:**
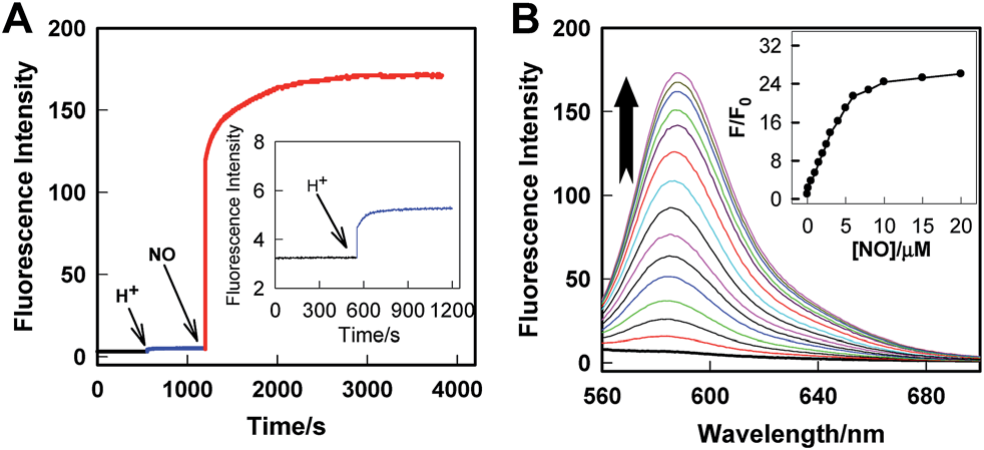
(A) Real-time fluorescence records of **Rhod-H-NO@MSN-CD** in a buffer solution (pH = 7.0), which was then treated with HCl solution, and a following addition of 20 μM NO. *λ*_ex_/*λ*_em_ = 550 nm/590 nm. (B) Fluorescence emission spectra of **Rhod-H-NO@MSN-CD** in the presence of different concentrations of NO in a buffer solution (pH = 5.0). The arrow indicates the signal changes as increases in the NO concentration. Inset: *F*/*F*_0_ of **Rhod-H-NO@MSN-CD** as a function of NO concentrations. Where *F*_0_ is the fluorescence intensity of **Rhod-H-NO@MSN-CD** at 590 nm and *F* is the fluorescence intensity of **Rhod-H-NO@MSN-CD** at 590 nm in the presence of NO.

The real-time kinetics of **Rhod-H-NO@MSN-CD** and its response toward NO in a buffer solution with different pH environments further confirmed that **Rhod-H-NO@MSN-CD** is pH-activatable for NO determination (Fig. 2A). After changing the neutral solution of **Rhod-H-NO@MSN-CD** to acidic conditions, there was slight, quick, increase in the fluorescence intensity which then remained steady over tens of minutes. However, the fluorescence intensity dramatically increased and reached a plateau in ∼30 min after being subsequently incubated with NO. When NO was first added into the neutral solution of **Rhod-H-NO@MSN-CD** and the solution was then adjusted to acidic pH, the fluorescence change further affirmed the pH activation characteristics (Fig. S11†). Then, we got the optimal pH range by measuring the fluorescence intensity enhancements of **Rhod-H-NO@MSN-CD** towards NO. As seen in Fig. S12,† a significant enhancement of *F*/*F*_0_ was observed over the pH range from 6.0 to 4.0, which is in good accord with lysosomal pH conditions, while **Rhod-NO@MSN-CD** was pH insensitive for NO response, suggesting that **Rhod-H-NO@MSN-CD** is a good candidate for the detection of lysosomal NO.

Next, we performed fluorescence titration studies of **Rhod-H-NO@MSN-CD** for NO in a buffer solution (pH = 5.0). As shown in Fig. 2B, the addition of NO with increasing concentrations from 0 to 20.0 μM NO, elicited a gradual enhancement of the emission band at 590 nm. The fluorescence enhancement (*F*/*F*_0_) reached a maximal value (26-fold) in the presence of 20.0 μM NO. Moreover, there was an excellent linear correlation between *F*/*F*_0_ and NO concentration in the range 0.2 to 6.0 μM with a detection limit (3*σ*/slope) of 100 nM, indicating that **Rhod-H-NO@MSN-CD** would be a potential tool to monitor endogenous NO in lysosomes. Moreover, **Rhod-H-NO@MSN-CD** exhibited high selectivity for NO with various biologically relevant species in an aqueous buffer solution at pH = 5.0 (Fig. S13†). The presence of reactive oxygen (H_2_O_2_, ·OH, O_2_^–^, ^1^O_2_, ClO^–^), reactive nitrogen (NO_2_^–^, NO_3_^–^, ONOO^–^), reactive sulfur (SO_3_^2–^, Cys, Hcy, GSH), and some common metal cations (K^+^, Na^+^, Ca^2+^, Mg^2+^, Zn^2+^, Fe^2+^, Cu^2+^), didn't cause an observable fluorescence enhancement, which was due to the specificity of the *o*-phenylenediamine receptor for NO.

**Fig. 3:**
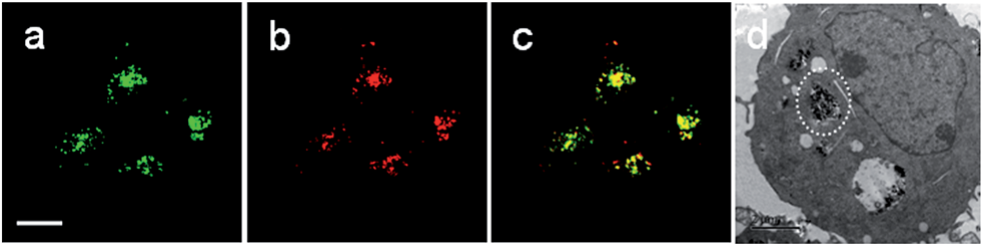
**Rhod-H-NO@MSN-CD** colocalizes to the lysosomes in HeLa cells. Cells were stained with: (a) 1.0 μM LysoTracker Green (*λ*_ex_ = 488 nm; *λ*_em_ = 505–530 nm) for 0.5 h at 37 °C, and (b) 50 μg mL^–1^**Rhod-H-NO@MSN-CD** (*λ*_ex_ = 550 nm; *λ*_em_ = 570–610 nm) with exogenous NO (100.0 μM) for 3.0 h at 37 °C. (c) Overlay of parts (a) and (b). Scale bar: 20 μm. (d) TEM micrograph of HeLa cells incubated with MSN-CD for 3.0 h.

**Rhod-H-NO@MSN-CD** was then investigated for the ability to both target the lysosomes and respond to NO in living cells. The cytotoxicities of **Rhod-H-NO@MSN-CD** on living cells were first evaluated by employing standard cell viability protocols (MTT assay) (Fig. S14†). After being cultured for 24 h, the cellular viability of HeLa cells was over 95% and no significant difference in the morphology was observed even when the concentration of **Rhod-H-NO@MSN-CD** was increased up to 100 μg mL^–1^, showing a very low cytotoxicity. Next, co-localization experiments were performed by co-staining HeLa cells with LysoTracker Green, MitoTracker Green and **Rhod-H-NO@MSN-CD**. From Fig. 3 and S15,† it can be seen that HeLa cells stained with **Rhod-H-NO@MSN-CD** for 2 h at 37 °C in the presence of 100 μM exogenous NO displayed significant red fluorescence, which merged well with the image from staining with LysoTracker Green but not with MitoTracker Green. Moreover, the intensity profiles of the linear regions of interest across the HeLa cells stained with **Rhod-H-NO@MSN-CD** and LysoTracker Green vary in close synchrony (Fig. S16†). The Pearson's colocalization coefficient and overlap coefficient were 0.865 and 1.528 respectively, as calculated using Autoquant X2 software, indicating that **Rhod-H-NO@MSN-CD** existed predominantly in the lysosomes and was activated by the coexistence of acidic pH and NO. TEM provides further additional physical evidence for the nanoparticles residence in the lysosomes (Fig. 3d). In view of the **Rhod-H-NO** probe selectively responding to the coexistence of H^+^ and NO *in vitro*, HeLa cells were pretreated with 1,8-bis(dimethylamino) naphthalene (DMAN), a common proton sponge, to promote lysosomal damage due to the proton-sponge effect.^18^ As expected, no obvious fluorescence was observed after incubation with **Rhod-H-NO@MSN-CD** in the presence of exogenous NO (Fig. S17†) because the probe escaped into the cytoplasm when the lysosomes were disrupted, further proving that the intracellular response behaviour of **Rhod-H-NO@MSN-CD** happened in acidic lysosomes rather than in cytoplasm and other organelles with neutral environment.

**Fig. 4:**
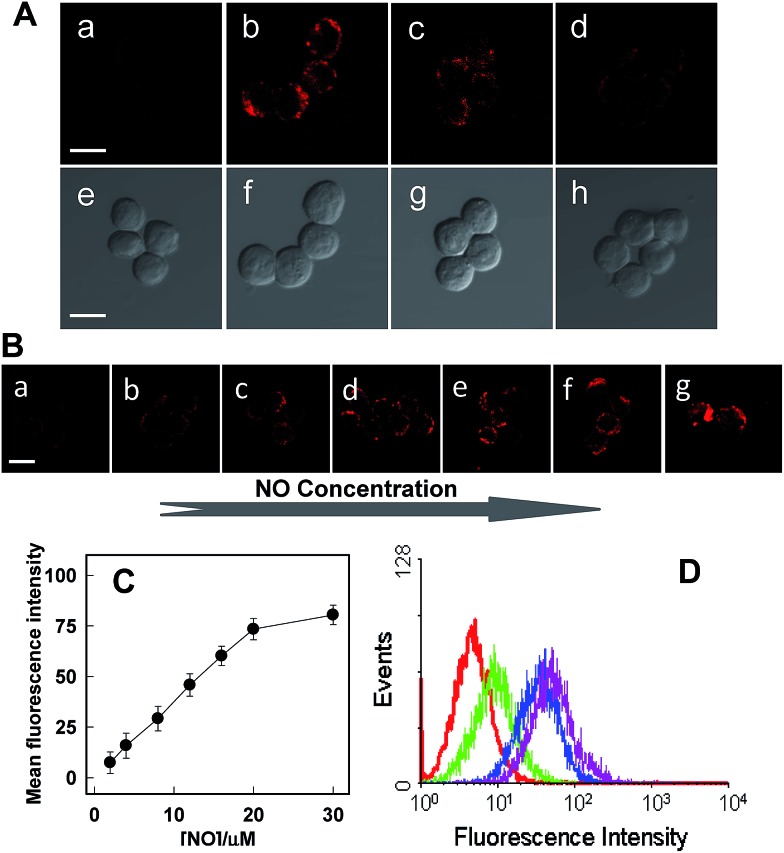
(A) Confocal microscopy images of endogenous NO of Raw 264.7 cells using **Rhod-H-NO@MSN-CD** under different conditions. (a, e) Cells incubated with the probe (50 μg mL^–1^) for 3.0 h. (b, f) Further treated (a) with NONOate (50 μM) for 20 min. (c, g) Cells were incubated with the probe (50 μg mL^–1^), l-Arg (5 mg mL^–1^), IFN-γ (400 U mL^–1^) and LPS (20 μg mL^–1^) for 12.0 h. (d, h) Cells were pretreated with l-Arg (5 mg mL^–1^), IFN-γ (400 U mL^–1^) and LPS (20 μg mL^–1^) for 12.0 h, then **Rhod-H-NO@MSN-CD** (50 μg mL^–1^) and PTIO were further incubated with the cells. (B) Raw 264.7 cells were incubated with different concentrations of NONOate (a–g 1.0, 2.0, 4.0, 6.0, 8.0, 10.0, 15.0 μM). (C) Normalized mean fluorescence intensity of images (a)–(g) *versus* different NO concentrations. (D) Flow cytometric analysis of Raw 264.7 cells loaded with **Rhod-H-NO@MSN-CD** (50 μg mL^–1^) and different stimulants for 12 h at 37 °C. Red line: cells loaded with **Rhod-H-NO@MSN-CD**. Pink line: cells loaded with **Rhod-H-NO@MSN-CD** and NONOate. Blue line: cells loaded with **Rhod-H-NO@MSN-CD**, l-Arg, IFN-γ and LPS. Green line: cells pretreated with PTIO, then loaded with **Rhod-H-NO@MSN-CD**, l-Arg, IFN-γ and LPS. Scale bar: 20 μm.

Next, further experiments were performed to verify the viability of **Rhod-H-NO@MSN-CD** to detect variations of NO level in living RAW 264.7 macrophages, which are well-known as NO producing cells. As shown in Fig. 4, the image of the probe-loaded macrophages gave barely detectable fluorescence (Fig. 4A(a)). Nevertheless, a significant increase in the intracellular fluorescence was visualized after further treatment with the widely used NO donor diethylamine NONOate (Fig. 4A(b)), and the intensity presented a tendency toward increased fluorescence over time and reached a maximum after 20 min (Fig. S18†), indicating that NO released from NONOate was able to diffuse into the lysosomes and then enter into the nanolab to react with **Rhod-H-NO**. According to the spontaneous NO releaser which in principle releases two equivalents of NO,^19^ the linearity between the relative fluorescence intensity and NO concentration was established, as shown in Fig. 4B and C, by pretreating the cells with different concentrations of NONOate, which demonstrated that the probe could quantitatively evaluate the intracellular NO content. Thus, to initiate physiological NO production, the well-known external stimuli, bacterial endotoxin lipopolysaccharide (LPS), l-arginine (l-Arg) and pro-inflammatory cytokine interferon-gamma (IFN-γ), were pretreated with cells for 12 h to promote NO production by inducible nitric oxide synthase (iNOS),^20^ and **Rhod-H-NO@MSN-CD** was subsequently introduced to the cells, showing a strong red fluorescence (Fig. 4A(c)). According to the linearity established in Fig. 4C, the average basal NO levels were estimated as *ca.* 8.36 μM. It should be confirmed that the fluorescence increase shown in Fig. 4A(c) was caused by NO generation and not by environmental changes. A NO scavenger, 2-(4-carboxyphenyl)-4,4,5,5-tetramethyl-imidazoline-1-oxyl-3-oxide (PTIO),^21^ was further introduced to the LPS and IFN-γ treated cells, which lead to the fluorescence being weakened obviously (Fig. 4A(d)). Moreover, we further quantitatively evaluated the endogenous NO in individual cells on a large scale using flow cytometry analysis. Fig. 4D demonstrates the intracellular accumulation of NO where an increase in intracellular fluorescence is indicated by a shift in the fluorescence signal measured in the FITC channel, which shows the same trend as the microscopy imaging of Fig. 4A. These results indicate that fluorescence imaging using **Rhod-H-NO@MSN-CD** as a nanolab is an effective tool for measuring different NO levels produced in lysosomes.

**Fig. 5:**
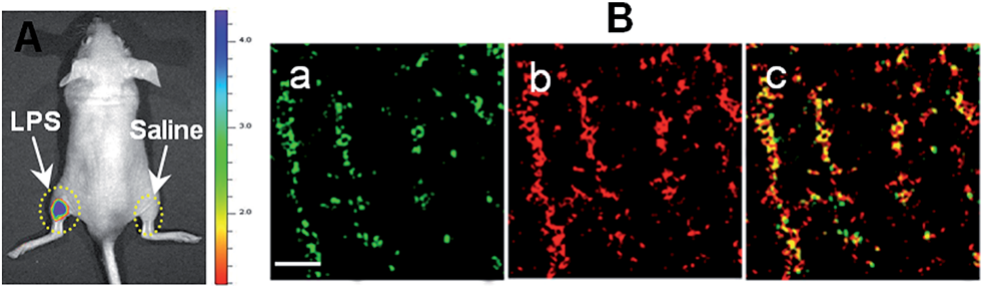
Detection of LPS-dependent NO generation in an inflamed mouse model using **Rhod-H-NO@MSN-CD**. (A) Fluorescence images of the mouse injected with **Rhod-H-NO@MSN-CD** during LPS-mediated inflammatory response *in vivo*. (B) Fluorescence images of the inflamed tissue slices. (a) CD11b, (b) **Rhod-H-NO@MSN-CD**, (c) the overlay of (a) and (b). Scale bar: 30 μm.

With an excellent lysosomal-targeting nanoprobe in hand, we used **Rhod-H-NO@MSN-CD** to investigate the produced NO level in lysosomes in an inflamed mouse model. 200 μL of LPS (1 mg mL^–1^) was subcutaneously injected into the left rear leg of a mouse to cause inflammation for 12 hours, while the right rear leg was treated with saline simply as the control. From *in vivo* imaging (Fig. 5A), one can see that strong fluorescence was observed from the left leg but not from the right leg after **Rhod-H-NO@MSN-CD** was intravenously injected, suggesting that the NO level increased significantly during inflammation. To further examine whether the signal came from the lysosomes of macrophage cells, the left leg skin was then sectioned to get the inflammation tissue. As shown in Fig. 5B, the fluorescence signal from **Rhod-H-NO@MSN-CD** was consistent with the results of the immune-staining histological sections with macrophage marker CD11b. From the results we came to the conclusion that the lysosomal NO level of macrophage cells during inflammation could be detected by **Rhod-H-NO@MSN-CD**.

## Conclusions

In conclusion, we have presented a novel strategy to fabricate a nanolab for the determination of intracellular bioactive molecules by engineering the highly selective and sensitive small molecular probe into mesoporous nanomaterials. The nanolab was capable of detecting lysosomal NO changes in the presence of exogenous or endogenous NO in living cells and *in vivo*. Significantly, this paper successfully addresses some challenges in intracellular imaging analysis. On the one hand, the location-activatable approach could effectively eliminate false signals and improve spatial resolution. On the other hand, the hermetical nanolab can protect the stability of the functional probe and avoid interference from a complex biological environment. In addition, the modification with β-CD on the surface of MSNs can improve the stability and biocompatibility of the nanomaterials in a physiological environment. In view of these merits, we anticipate that this design strategy would open up a new train of thought for the development of efficient molecular tools for bioanalytical and biomedical applications.

## Supplementary Material

Supplementary information
