# Supplementary material for: Engineering a nanolab for the determination of lysosomal nitric oxide by the rational design of a pH-activatable fluorescent probe
Source: Chem Sci. 2015 Nov 30;7(3):1920–5. doi: 10.1039/c5sc04415d (PMC5966799; doi:10.1039/c5sc04415d)
Supplement: Supplementary file 1 [file SC-007-C5SC04415D-s001.pdf]

Electronic Supplementary Material (ESI) for Chemical Science.
This journal is © The Royal Society of Chemistry 2015

Engineering a Nanolab for Determination of Lysosomal Nitric Oxide by Rational Design of a pH-activatable Fluorescent Probe

Yinhui Li,*[,a] Wei Wu,[a] Jinfeng Yang,[c] Lin Yuan,[a] Changhui Liu,[a] Jing Zheng,[a] and Ronghua Yang,*[,a, b]

[a] State Key Laboratory of Chemo/Biosensing and Chemometrics, College of Chemistry and Chemical Engineering, Hunan University, Changsha, 410082, China. [b] School of Chemistry and Biological Engineering, Changsha University of Science and Technology, Changsha, 410004, China. [c] Tumor Hospital, Xiangya School of Medicine, Central South University, Changsha, 410013, China.

To whom correspondence should be addressed:

E-mail: yinhuili16@163.com; yangrh@pku.edu.cn

Fax: +86-731-8882 2523

Materials and Apparatus. All solvents and other chemicals were of analytical reagent grade, and were used without further purification unless otherwise stated. Water used in all experiments was doubly distilled and purified by a Mili-Q system (Milipore, USA). HeLa cells (cervical cancer cells), Raw264.7 cells (normal human immune cells) were obtained from the cell bank of the Central Laboratory at Xiangya hospital (Changsha, China) and were cultured in Dulbecco's Modified Eagle Medium(DMEM), supplemented with 10% fetal bovine serum, 1% penicillin, and 1% streptomycin at 37 °C in a 5% CO_2/95% air incubator. Nude mice (about 18g in weight) were purchased from SJA Co., Ltd. (Changsha, China). All animal operations were in accord with institutional animal use and care regulations, according to protocol No. SYXK (Xiang) 2008-0001, approved by Laboratory, approved by Laboratory Animal Center of Hunan. Working solutions were prepared by successive dilution of the stock solution with Britton−Robinson buffer solution.

Nuclear magnetic resonance spectra were recorded at 400 MHz and carbon spectra were recorded at 100 MHz on an Invoa-400 spectrometer. Mass spectra were obtained on LCQ/Advantage HPLC-Mass spectrotometer. Energy-dispersive X-ray (EDX) analysis was carried out using a HITACHI S-4500 instrument. The transmission electron microscopic (TEM) images were obtained on a JEM-2100 transmission electron microscope (JEOL Ltd., Japan). Nitrogen adsorption/desorption measurement was obtained through Brunauer-Emmett-Teller (BET, NOVA 2200e, Quanthachrome, USA). Zeta potential analysis was performed on a Zetasizer (Nano-Z, Malvern, UK). Fourier transform infrared (FTIR) spectra were obtained from a TENSOR 27 spectrometer (Bruker Instruments Inc., Germany). Fluorescence emission spectra were measured on a PTI QM4 fluorescence system (Photo technology international, Birmingham, NJ). Fluorescence images of cells and tissue slices were obtained using an Olympus FV1000-

MPE multiphoton laser scanning microscope (Japan). Fluorescent images of mice were taken by a Caliper VIS Lumina XR small animal optical in vivo imaging system (USA). UV-Vis absorption spectra were recorded in 1.0 cm path length quartz cuvettes on a Hitachi U-4100 UV/Vis spectrometer (Kyoto, Japan). pH was measured by model 868 pH meter (Orion).

Synthesis of Rhod-NO.[1] Rhodamine B (500 mg, 0.76 mmol) was dissolved in CH_2Cl_2 (5.0 ml) at room temperature, and then 1-hydroxypyrrolidine-2, 5-dione (262.2 mg, 2.28 mmol) and DCC (313.2 mg, 1.52 mmol) were added. The mixture was allowed to react for 10 h, and subsequently triethylamine (0.7 mL) and o-diaminobenzene (240 mg, 2.22 mmol) were added. After stirring for 3 h, the mixture was concentrated under vacuum and the crude product was purified by silica column chromatography (CH_2Cl_2:CH_3OH = 100:1) to give **Rhod-NO** as a white powder (170 mg, 46.4% yield).

Synthesis of Rhod-H-NO. Rhod-NO (120mg, 0.225 mmol) and propionaldehyde (170μL) was dissolved in CH_3OH (5.0 mL) in room temperature, and then several drops of HOAc was added. The mixture was refluxed overnight under N_2 atomsphere. The solvent was removed by evaporation under reduced pressure. The crude residue was purified by silica gel column chromatography using CH_2Cl_2 to give **Rhod-H-NO** as a white powder (78mg, 65.2% yield). ^1H NMR (400 MHz, $CDCl_3$) δ 8.02 (d, J = 8.0 Hz, 1H), 7.55 (t, J = 6.0 Hz, 2H), 7.25 (s, 1H), 6.94 (t, J = 8.0 Hz, 1H), 6.63 (d, J = 8.0 Hz, 2H), 6.54 (d, J = 4.0 Hz, 1H), 6.41 (t, J = 8.0, 1H), 6.31 (d, J = 8.0 Hz, 2H), 6.08 (d, J = 8.0 Hz, 1H), 4.30 (t, J = 6.0 Hz, 2H), 3.31 (dd, J = 8.0 Hz, J = 8.0 Hz, 8H), 1.91 (dd, J = 4.0 Hz, J = 4.0 Hz, 2H), 1.13 (t, J = 6.0 Hz, 12H), 0.87 (t, J = 8.0 Hz, 3H); ^{13}C NMR (100 MHz, $CDCl_3$) δ 12.5, 14.1, 24.9, 25.6, 29.7, 34.0, 44.3, 49.1, 77.3, 98.0, 108.0, 116.9, 118.2, 122.1, 123.4, 124.3, 129.3, 130.0, 131.9, 132.6, 144.5, 149.0, 152.3, 154.0, 156.9, 166.4. MS (ESI) m/z calcd for $C_{37}H_{40}N_4O_2$ 572.3, found 573.1.

The synthesis of *β*-CD-alkyne:[2] A suspension of *β*-CD hydrate (11.5 g, 10 mmol) and Ts_2O (4.9 g, 15 mmol) in 250 mL of water was stirred at room temperature for 2 h. A solution of NaOH (5.0 g in 50 mL of H_2O) was added, and after 10 min unreacted Ts_2O was removed by filtration through a sintered glass funnel. The filtrate was brought to pH ~8 by the addition of NH_4C1 (13.4 g), affording p-toluenesulfonic acid-substituted cyclodextrin (CD-TSO) as a precipitate that was collected after cooling at 4 °C overnight; yield, 4.0 g (27%). The CD-TSO (856.4 mg, 4.98 mmol) was added into 2-propynylamine (1.8 mL). The mixture was stirred at 45 ^{o}C for 24 h under nitrogen atmosphere. Subsequently, the mixture was added with acetonitrile (8.5 mL) and filtrated to give the crude product, which was further purified by recrystallization from methanol aqueous solution (CH_3OH:H_2O=1:1). The resulting residue was filtrated and repeated the recrystallization step to afford white crystalline solid (778 mg, 90% yield).

The synthesis of MSN-N_3. The as-synthesized MSN (0.50 g) was refluxed for 20 h in 40.0 mL of anhydrous toluene with 3-Chloropropyltrimethoxysilane (0.50 mL) to yield the 3-chloropropyl-functionalized MSN (MSN-Cl) material. The MSN-Cl nanoparticles were then collected by centrifugation, washed thoroughly with toluene and ethanol, and dried in vacuum. To remove the surfactant template (CTAB), MSN-Cl particles (0.40 g) were refluxed for 6 h in an ethanolic solution (70 mL) containing HCl (0.70 mL, 37.2%). The resulting material was separated by centrifugation and extensively washed with nanopure water and ethanol. The surfactant-free MSN-Cl material was placed under high vacuum with heating at 333 K to remove the remaining solvent from the mesopores. The MSN-Cl (0.20 g) was added to a saturated solution of sodium azide in N,N-dimetylformamide (DMF, 20 mL) solution and stirred at 90 °C for 12 h. The resulting mixture was separated by centrifugation, redispersed in PBS buffer solution, and stirred for 6 h to remove remaining DMF from the mesopores. After purification,

the azide-functionalized nanoparticles (MSN-N_3) were washed with water and ethanol before being dried in vacuum.

Preparation of Rhod-H-NO@MSN-CD: MSN-N_3 (10 mg) and **Rhod-H-NO** (53.4 mg) were placed in a round-bottom flask containing MeOH (5 mL). The solution mixture was stirred at room temperature for 24 h to allow the **Rhod-H-NO** to be encapsulated by the MSN-N_3. The resulting particles were then separated by centrifugation and dispersed in H_2O (1 mL) with CD-alkyne (35 mg), $CuSO_4{\cdot}5H_2O$ (2.75 mg, 11 mmol), TBAB (10mg) and sodium ascorbate (10.9 mg, 55mmol). The mixture was stirred at room temperature for 3 days. The resulting particles were then filtered and purified by centrifugation and washing with H_2O/EtOH.

Spectrophotometric Measurements. Nitric oxide (NO) was generated from DEA/NONOate. A stock solution of DEA/NONOate (1 mM) was prepared in 0.01M NaOH solution. Both the UV/vis absorption and fluorescence measurements of **Rhod-H-NO** were conducted in Britton−Robinson buffer solution. 1.0 mL buffer solution (pH 5.0) containing 1.0 μM **Rhod-H-NO** was first introduced to a quartz cell. Following the additions of different concentration of DEA/NONOate solution, the fluorescence intensities were recorded at excitation wavelength of 550 nm with an emission wavelength range from 560 to 750 nm.

To measure leakage of **Rhod-H-NO** from MSNs, **Rhod-H-NO@MSN** and **Rhod-H-NO@MSN-CD** were suspended in 500 μL of buffer solution, respectively, sealed in a home-made dialysis tube with a molecular weight cutoff of 8000-14000 Da, and then immersed in 1.5 mL of buffer solution containing 50.0 μM NO. The system was moderately shaken. The fluorescence intensity of dialysis fluid was measured at certain time points.

Cell Cytotoxic Assays and Flow Cytometry (FCM). First, the cytotoxic effect of **Rhod-H-NO@MSN-CD** were assessed using the MTT assay. Raw 264.7 cells were seeded into 96-well

plates and grown for 48 hours. These cells were then incubated with **Rhod-H-NO@MSN-CD** at different concentrations for 24 h. Afterwards, cells were incubated in media containing 0.5 mg mL^{-1} of MTT for 2 h. The precipitated formazan violet crystals were dissolved in 150 μL of dimethyl sulfoxide (DMSO) at 37 °C. The absorbance was measured at 490 nm by a multidetection microplate reader.

For FCM studies, macrophages cells RAW 264.7 were divided into four groups, group one were treated with Rhod-H-NO@MSNs-CD (50μg/mL) in 200 μL of cell culture medium for 12 h, group two were treated with **Rhod-H-NO@MSN-CD** and NONOate (50 μM) for 12 h, group three were treated with **Rhod-H-NO@MSN-CD**, L-Arg (5 mg/mL), IFN-γ (400 U/mL) and LPS (20 μg/mL) for 12 h, group four were pretreated with PTIO, then loaded with **Rhod-H-NO@MSN-CD**, L-Arg (5 mg/mL), IFN-γ (400 U/mL) and LPS (20 μg/mL) for 12 h. All the cells were washed with three times with PBS, suspended in 200 μL of binding buffer, and subjected to flow cytometry analysis using a BD FACSCalibur cytometer by counting 10 000 events.

Cell Imaging. Before detection of the intracellular NO, the co-localization experiments were performed firstly. The HeLa cells were incubated with **Rhod-H-NO@MSN-CD** (50 μg/mL) and DEA/NONOate (50 μM) for 2 hour at 37 ℃ with 5% CO_2, and then Lysosome-tracker Lyso Tracker Green (1 μM) were further incubated for 30 min at the same condition. Finally, the cells were washed three times with phosphate buffered saline (PBS) before imaging. (**Rhod-H-NO@MSN-CD** channel: λ_{ex}=559 nm, λ_{em}=570–600 nm bandpass. LysoTracker Green channel: λ_{ex}=488 nm, λ_{em}=505–530 nm bandpass. Yellow: colocalization of red and green fluorescence).

For cells imaging of exogenous NO in lysosome, the cells were pretreated with **Rhod-H-NO@MSN-CD** (50 μg/mL) for 2h before being washed three times with PBS, then incubated

with different concentrations of DEA/NONOate. For cells imaging of endogenous NO in lysosome, the cells were incubated with **Rhod-H-NO@MSN-CD** in the absence or presence of L-arginine (L-Arg, 0.5 mg/mL), interferon-r (IFN-r, 150 units/mL), Lipopolysaccharide (LPS, 20μg/mL) for 12 h. After washing with PBS buffer, the cells were subjected to imaging analysis. Fluorescence images (570-600 nm) were obtained by excitation with a multi Ar laser (559 nm).

Animal Models and in Vivo Animal Study. Before animal imaging, nude mouse were starved for 12 h to rule out the possible of food fluorescence interference at the emission band of **Rhod-H-NO**. After anesthesia, 200 μL of 1 mg/mL LPS was injected on left rear leg of mouse, and the right rear leg of mice was injected 200 μL saline as control experiment. After 12 h, 200 μL of **Rhod-H-NO@MSN-CD** (500 μg/mL) was then injected through tail vein. 30 min later, anesthesia of the mice was induced and maintained by inhalation of 5% isoflurane in 100% oxygen. Then, the mouse was placed into the imaging chamber, and kept under anesthetic using an isoflurane gas anesthesia system. Whole body images were acquired using a Caliper VIS Lumina XR small animal optical in vivo imaging system. The imaging mode is set as excitation scan and Input/Em was chosen as 535 nm for excitation, 575-650 nm for emission channels, respectively. All fluorescence images were acquired with auto exposure (FOV, 10cm; f/stop2; bin, high resolution), and fluorescence intensity was scaled as units of photons per second per centimeter square per steradian (ps^{-1} cm^{-2} sr^{-1}).

Tissue Slices Imaging Studies. After imaging in vivo, the skin of the inflamed (left rear) tissues were harvested and embedded in tissue-freezing medium, frozen and consecutively cryo-sectioned into 10 μm slices. Then, the slices were washed with PBS three times and fixed with 4% paraformaldehyde for 20 min at 37 ^{o}C. To confirm the stain pattern of **Rhod-H-NO@MSN-CD**, rat anti mouse CD11b antibody (Abcam, dilution factor 1:300) was applied and was visualized by

cy5-conjugated goat anti rat secondary antibody (Life TechnologiesTM, dilution factor 1:300). Fluorescence images were obtained by Olympus FV1000-MPE multiphoton laser scanning microscope. Fluorescence images were collected in two channels (green: 570-600 nm; red: 640-680 nm) upon excitation with a multi Ar laser (559 nm and 630nm).

Transmission Electron Microscopy Analysis. HeLa cells were treated with **Rhod-H-NO@MSN-CD** in media for 3.0 h at 37 °C. After washing with PBS, the cells were detached and centrifuged. The cell pellets were fixed with 2.5% glutaraldehyde in 0.1 M phosphate buffer for 2.5 h, dehydrated using an ascending alcohol series (20, 40, 60, 80 and 100% twice) for 20 min for each change, and then embedded in Araldite resin at 65 °C overnight. A 70 nm section was placed on a TEM grid and stained with saturated uranyl acetate and 0.2% Reynolds lead citrate before TEM imaging. Images were collected using a JEOL 1200 EXII scanning and transmission electron microscope (Japan Electron Optics Laboratory, Peabody, MA).

References:

1. L. Yuan, W. Lin, Y. Xie, B. Chen, S. Zhu, *J. Am. Chem. Soc.* **2012**, *134*, 1305-1315.

2. Z. Guo, Y. Jin, T. Liang, Y. Liu, Q. Xu, X. Liang, A. Lei, *J. Chromatogr. A* **2009**, *1216*, 257-263.

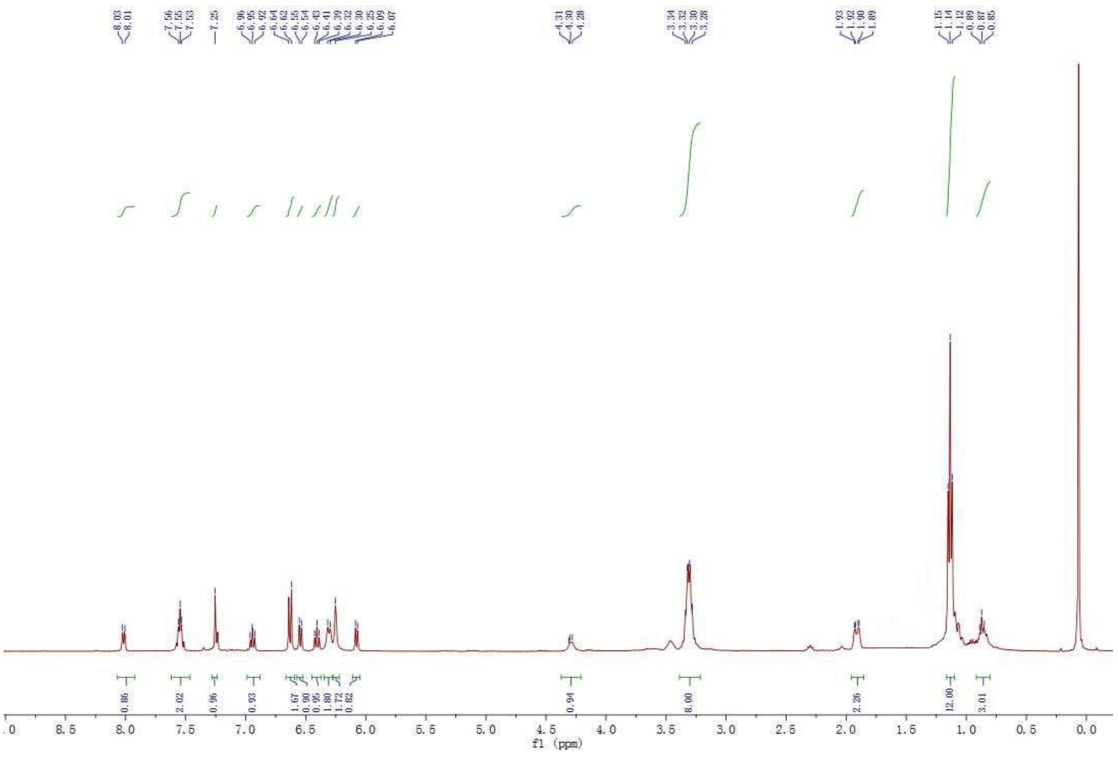

Fig. S1. ^{1}H NMR of **Rhod-H-NO**.

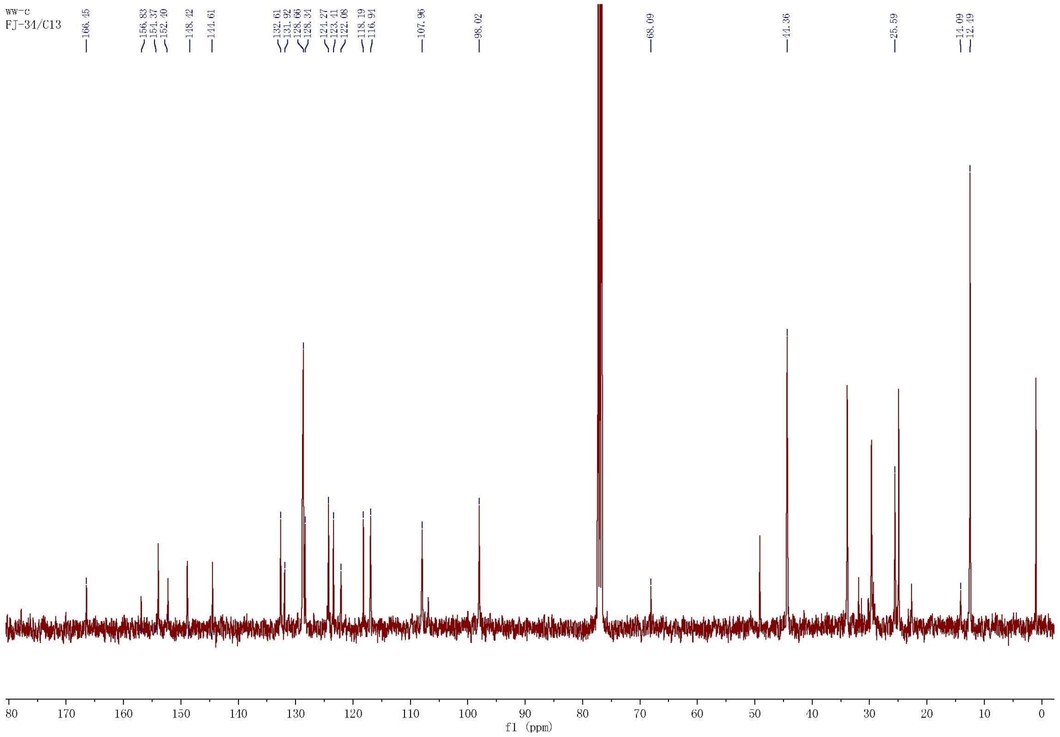

Fig. S2. ^{13}C NMR of **Rhod-H-NO**.

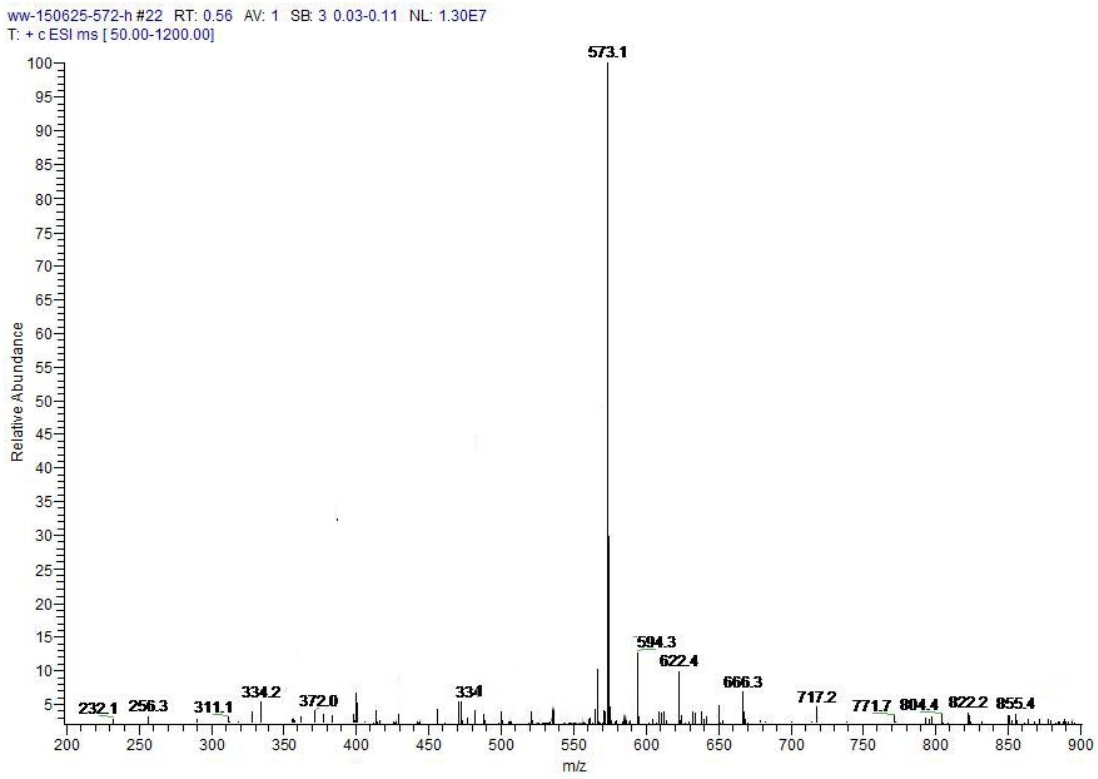

Fig. S3. ESI-MS of **Rhod-H-NO**.

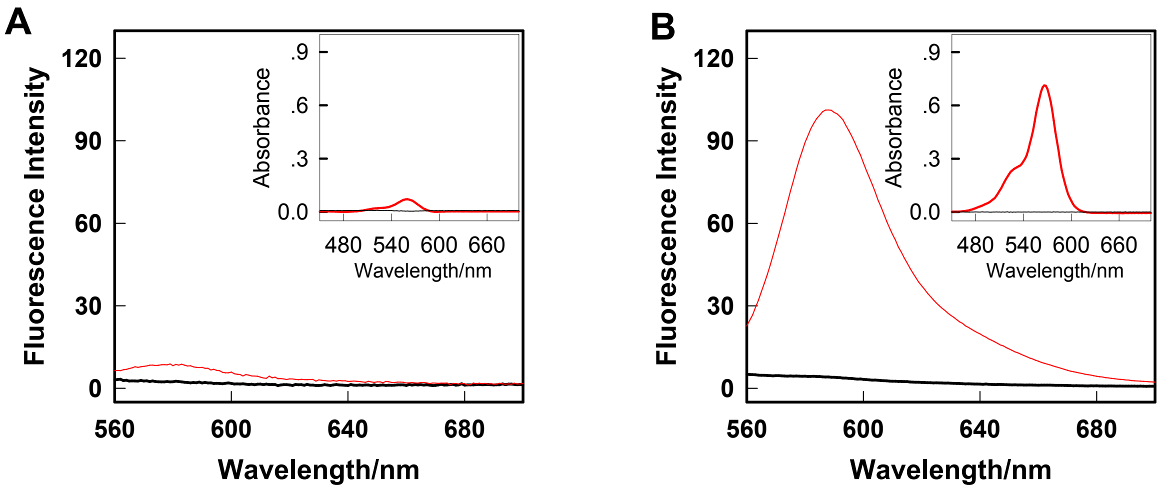

Fig. S4. Fluorescence emission spectra of **Rhod-H-NO** in the absence (black line) and presence (red line) of NO in buffer solution containing 10% CH_3CN. (A) pH 7.0, (B) pH 5.0. The inset is the absorption spectra corresponding to the fluorescence emission spectra.

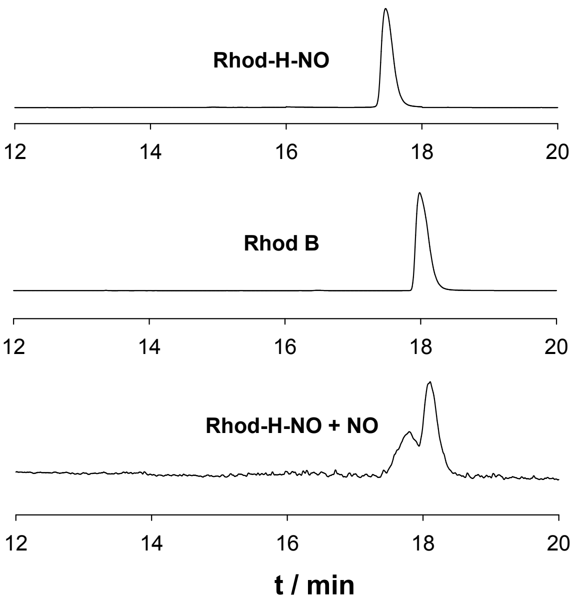

Fig. S5. HPLC analysis of probe **Rhod-H-NO**, compound **Rhod B** and the reaction product of **Rhod-H-NO** with NO in buffer solution (pH 5.0).

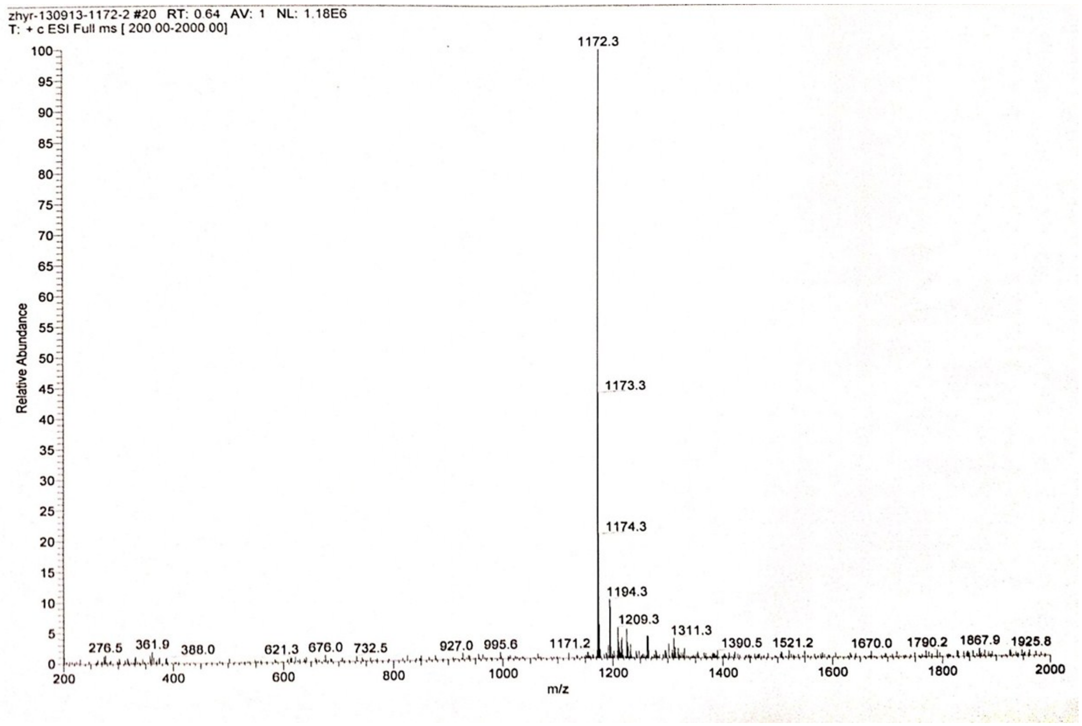

Fig. S6. ESI-MS of ***β*-CD-alkyne**.

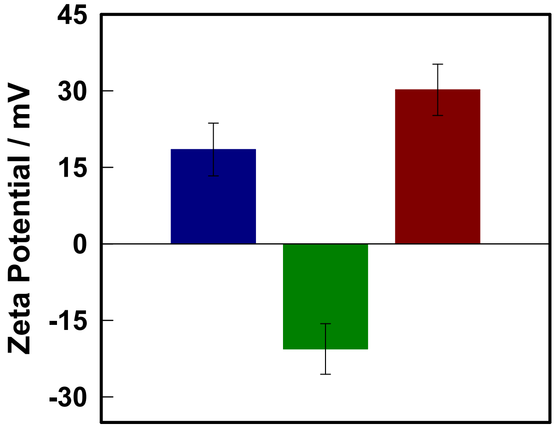

Fig. S7. Zeta potentials of MSN-Cl (blue bar), MSN-N_3 (green bar) and MSN-CD (red bar).

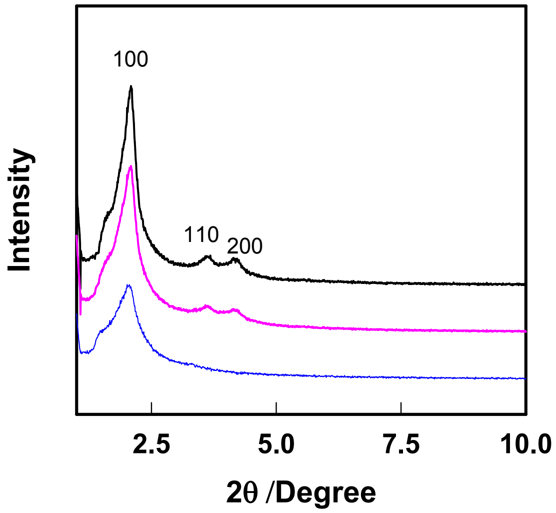

Fig. S8. Small-angle powder XRD patterns of MSN-Cl (black line), MSN-CD (pink line) and Rhod-H-NO@MSN-CD (blue line).

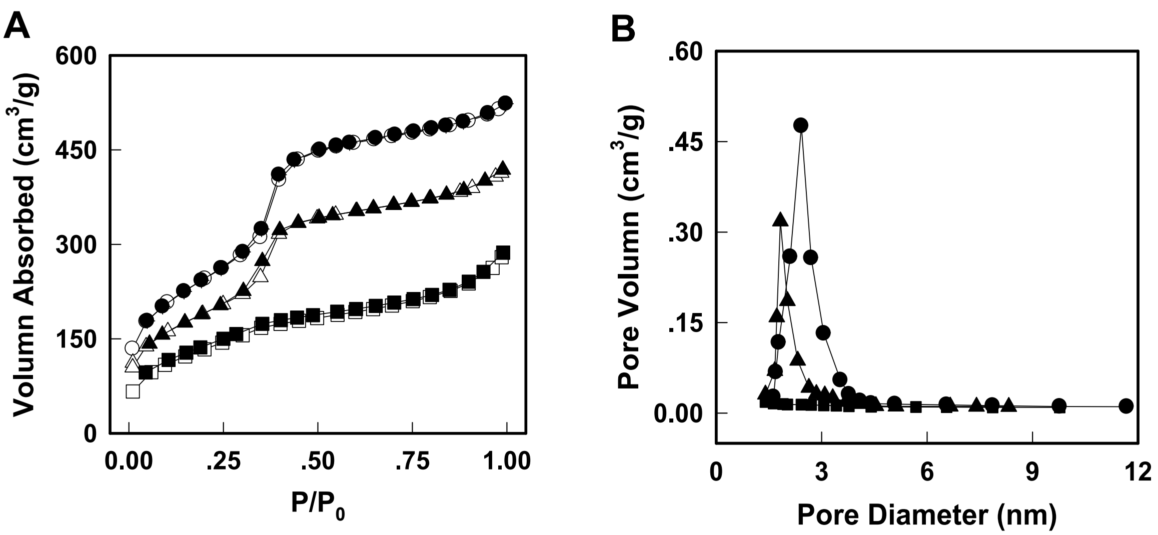

Fig. S9. (A) Nitrogen adsorption-desorption isotherms and (B) Pore size distributions of MSN-Cl (●), MSN-CD (▲) and **Rhod-H-NO@MSN-CD** (■).

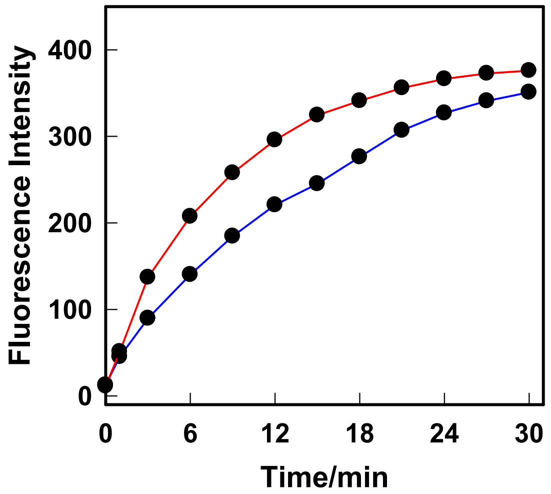

Fig. S10. Real-time fluorescence intensity records of 50 μg/mL **Rhod-H-NO@MSN** (red line) and **Rhod-H-NO@MSN-CD** (blue line) upon addition of NO in buffer solution (pH 5.0). $\lambda_{ex}/\lambda_{em}$ = 550 nm/590 nm.

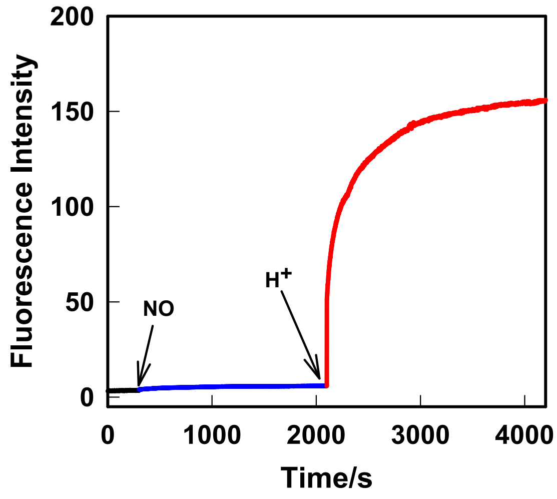

Fig. S11. Real-time fluorescence records of **Rhod-H-NO@MSN-CD** buffer solution (pH 7.0), then addition of 20 μM NO to the solution, finally, treated the solution with HCl solution. $\lambda_{ex}/\lambda_{em}$ = 550 nm/ 590nm.

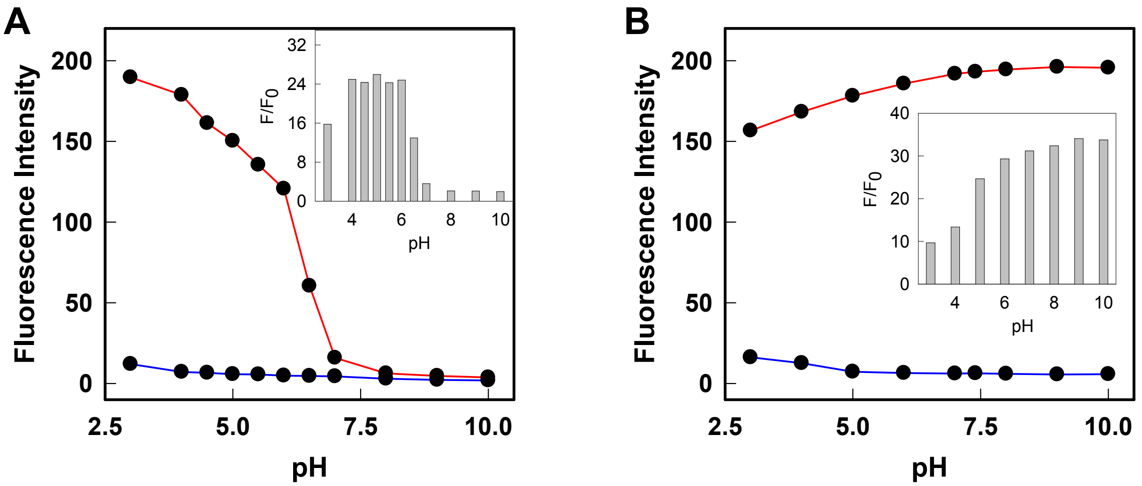

Fig. S12. Effect of pH on the fluorescence emission of **Rhod-H-NO@MSN-CD** (A) and **Rhod-NO@MSN-CD** (B) in the absence (blue line) and presence of 50.0 μM NO (red line). Inset: F/F_0 of **Rhod-H-NO@MSN-CD** and **Rhod-NO@MSN-CD** as a function of pH.

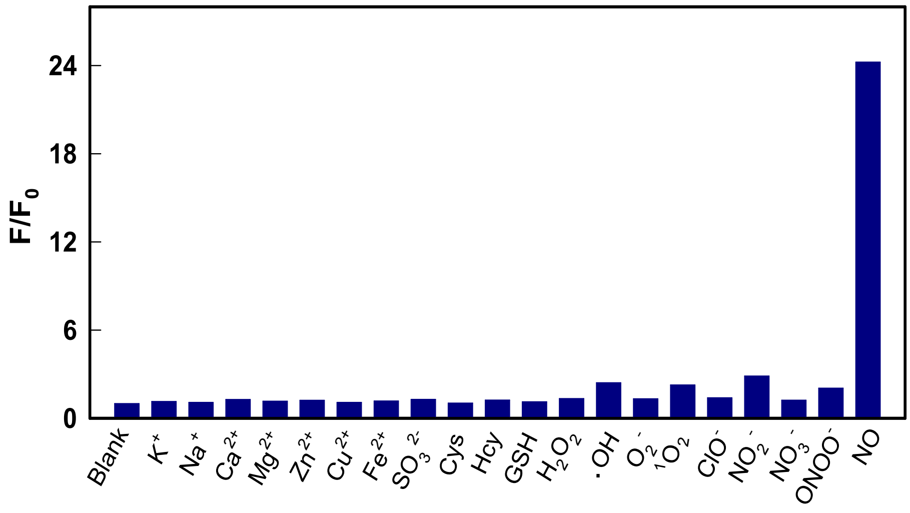

Fig. S13. Fluorescence responses of **Rhod-H-NO** (1 μM) towards various analytes. K^+, Na^+, Ca^{2+}, Mg^{2+}, Zn^{2+}, Fe^{2+}, Cu^{2+} /5 mM, SO_3^{2-}, Cys, Hcy, GSH /5 mM, H_2O_2, ·OH, O_2^-, $^1O_2$, ClO^-, NO_2^-, NO_3^-, $ONOO^-$/100 μM.

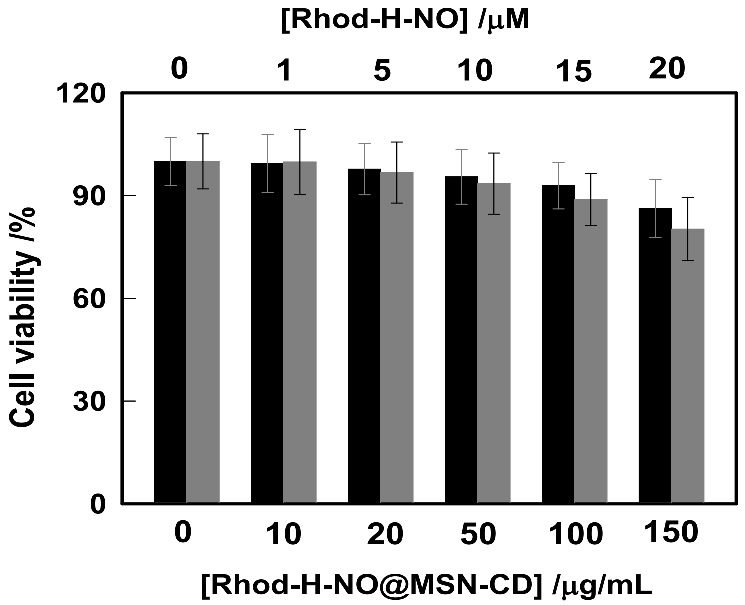

Fig. S14. Cell viability values (%) estimated by MTT proliferation. HeLa cells were incubated with 0−150 $\mu g \cdot mL^{-1}$ **Rhod-H-NO@MSN-CD** (black bars) and 0-20 μM **Rhod-H-NO** (grey bars)

at 37 ºC for 24 h, respectively. Cells without addition of **Rhod-H-NO@MSN-CD** and **Rhod-H-NO** were taken as the control experiment, and the viability was set as 100%.

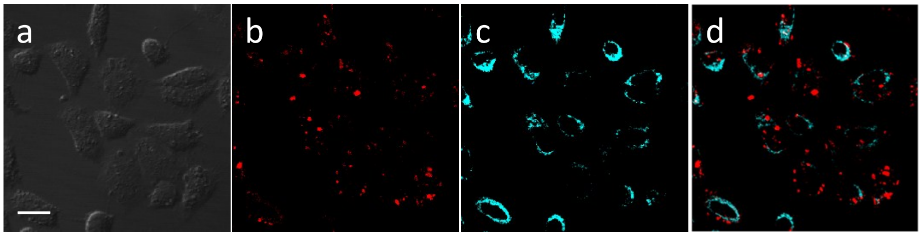

Fig. S15. Rhod-H-NO@MSN-CD colocalizes to the lysosome in HeLa cells. (a) Bright filed. (b) Cells were stained with 50 μg/mL **Rhod-H-NO@MSN-CD** with exogenous NO (100.0 μM) for 3.0 h at 37 °C. (c) Cells were stained with 2.0 μM mitochondria Tracker for 0.5 h at 37 °C, and (d) Overlay of parts b and c. Scale bar: 20 μm.

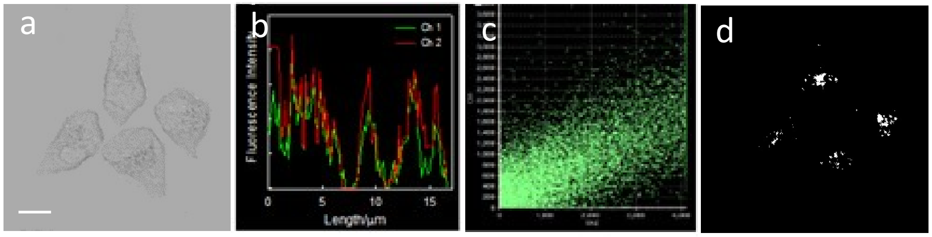

Fig. S16. Rhod-H-NO@MSN-CD colocalizes to the lysosome in HeLa cells. These images correspond to **Figure 3** in main text. (a) Bright filed. (b) Intensity profile of region of interest (ROI) across the HeLa cell. (c) Intensity scatter plot of LysoTracker Green and **Rhod-H-NO@MSN-CD**. (d) Simulated image (the white pixels, as represented by the scatter plot (c), were highlighted by selecting the points with a red rectangular selection.). Scale bar: 20 μm.

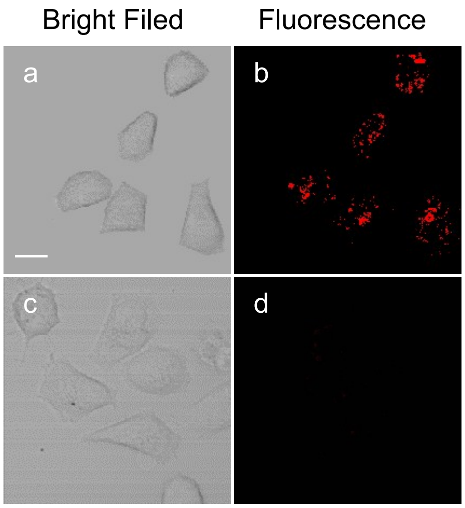

Fig. S17. Confocal microscopy images of HeLa cells using **Rhod-H-NO@MSN-CD** under different conditions. (a,b), Cells incubated with **Rhod-H-NO@MSN-CD** (50 μg/mL) for 3.0 h. (c,d) Cells were first treated with with proton sponge of DMAN for 2.0 h, then incubated with **Rhod-H-NO@MSN-CD** for 3.0 h. λ_{ex} = 559 nm; λ_{em} = 570-600 nm. Scale bar: 20 μm.

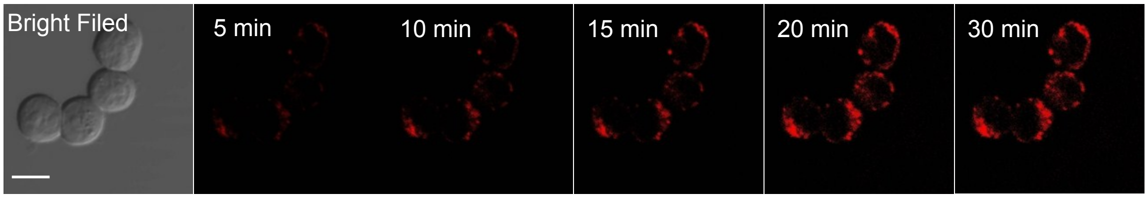

Fig. S18. Time based fluorescence images. RAW264.7 macrophages were pretreated with 50 μg/mL **Rhod-H-NO@MSN-CD** for 3 hours, after washing with PBS for three times, the cells were further incubated with 50 μM NONOate for real-time imaging analysis. λ_{ex} = 559 nm; λ_{em} = 570-600 nm. Scale bar: 20 μm.
